# Financial Strain Among Mexican American Adult Children Caregivers Living With Aging Parents

**DOI:** 10.1093/geroni/igaf122.599

**Published:** 2025-12-31

**Authors:** Anna Bokun, Flavia Andrade, Jacqueline Angel, Phillip Cantu, Sunshine Rote

**Affiliations:** The University of Texas at Austin, Boston, Massachusetts, United States; University of Illinois--Urbana-Champaign, Urbana, Illinois, United States; The University of Texas at Austin, Austin, Texas, United States; UTMB Health, The University of Texas Medical Branch, Galveston, Texas, United States; University of Louisville, Louisville, Kentucky, United States

## Abstract

As more individuals provide care to aging parents, the question arises of whether adult children who provide care to and live with their parents experience increased financial strain and how this might differ if the caregiver is the head of the household. Despite the cost-sharing benefits of co-residing, adult child caregivers may face greater financial strain due to out-of-pocket costs, medical expenses, and home modifications. We use the Hispanic Established Populations for the Epidemiologic Study of the Elderly (HEPESE) data (2010–11), a survey of Mexican-Americans in five southwestern states in the United States, supplemented with nationally representative data from the American Community Survey. Our dependent variable is an indicator of caregiver financial strain developed from an index of five measures of financial difficulty in meeting financial expenses. Logistic regressions show that co-residing more than doubled the odds of financial strain (OR 2.17, 95% CI [1.22, 3.82], p < 0.01), especially if the caregiver is younger or late-middle aged. We find no observed association between household headship status and financial strain. This study provides empirical evidence for public policies that reduce family caregivers’ financial burden through direct compensation and expanded support services.

